# Quantitative proteomics comparison of arachnoid cyst fluid and cerebrospinal fluid collected perioperatively from arachnoid cyst patients

**DOI:** 10.1186/2045-8118-10-17

**Published:** 2013-04-29

**Authors:** Magnus Berle, Ann Cathrine Kroksveen, Hilde Garberg, Mads Aarhus, Øystein Ariansen Haaland, Knut Wester, Rune Johan Ulvik, Christian Helland, Frode Berven

**Affiliations:** 1 Department of Clinical Science, University of Bergen, Bergen, Norway; 2 Proteomics Unit (PROBE), Department of Biomedicine, University of Bergen, Bergen, Norway; 3 Department of Clinical Medicine, University of Bergen, Bergen, Norway; 4 Department of Urology, Surgical Clinic, Haukeland University Hospital, Bergen, Norway; 5 The KG Jebsen Centre for MS-research, Department of Clinical Medicine, University of Bergen, Bergen, Norway; 6 Department of Neurosurgery, Oslo University Hospital, Oslo, Norway; 7 Centre for Medical Genetics and Molecular Medicine, Haukeland University Hospital, Haukeland, Norway; 8 Department of Public Health and Primary Health Care, University of Bergen, Bergen, Norway; 9 Department of Neurosurgery, Haukeland University Hospital, Haukeland, Norway; 10 Laboratory of Clinical Biochemistry, Haukeland University Hospital, Haukeland, Norway; 11 The Norwegian Multiple Sclerosis Competence Centre, Haukeland University Hospital, Haukeland, Norway

## Abstract

**Background:**

There is little knowledge concerning the content and the mechanisms of filling of arachnoid cysts. The aim of this study was to compare the protein content of arachnoid cysts and cerebrospinal fluid by quantitative proteomics to increase the understanding of arachnoid cysts.

**Methods:**

Arachnoid cyst fluid and cerebrospinal fluid from five patients were analyzed by quantitative proteomics in two separate experiments.

In a label-free experiment arachnoid cyst fluid and cerebrospinal fluid samples from individual patients were trypsin digested and analyzed by Orbitrap mass spectrometry in a label-free manner followed by data analysis using the Progenesis software.

In the second proteomics experiment, a patient sample pooling strategy was followed by MARS-14 immunodepletion of high abundant proteins, trypsin digestion, iTRAQ labelling, and peptide separation by mix-phase chromatography followed by Orbitrap mass spectrometry analysis. The results from these analyzes were compared to previously published mRNA microarray data obtained from arachnoid membranes.

**Results:**

We quantified 348 proteins by the label-free individual patient approach and 1425 proteins in the iTRAQ experiment using a pool from five patients of arachnoid cyst fluid and cerebrospinal fluid. This is by far the largest number of arachnoid cyst fluid proteins ever identified, and the first large-scale quantitative comparison between the protein content of arachnoid cyst fluid and cerebrospinal fluid from the same patients at the same time. Consistently in both experiment, we found 22 proteins with significantly increased abundance in arachnoid cysts compared to cerebrospinal fluid and 24 proteins with significantly decreased abundance. We did not observe any molecular weight gradient over the arachnoid cyst membrane. Of the 46 proteins we identified as differentially abundant in our study, 45 were also detected from the mRNA expression level study. None of them were previously reported as differentially expressed. We did not quantify any of the proteins corresponding to gene products from the ten genes previously reported as differentially abundant between arachnoid cysts and control arachnoid membranes.

**Conclusions:**

From our experiments, the protein content of arachnoid cyst fluid and cerebrospinal fluid appears to be similar. There were, however, proteins that were significantly differentially abundant between arachnoid cyst fluid and cerebrospinal fluid. This could reflect the possibility that these proteins are affected by the filling mechanism of arachnoid cysts or are shed from the membranes into arachnoid cyst fluid. Our results do not support the proposed filling mechanisms of oncotic pressure or valves.

## Background

Arachnoid cysts (AC) are congenital malformations of the arachnoid; a benign malformation with reported prevalence of up to 1.1% [1,2]. The mechanism of formation of such cysts is not known, although several studies have tried to investigate and understand the biological basis of AC [3-11]. Anatomically, ACs originate from splitting of the arachnoid mater (AM), thus ACs are truly intra-arachnoid in nature [9,10]. True ACs are considered to be developmental or congenital mistakes of the arachnoid architecture [12]. By electron microscopy, Rengachary *et al.*[10] observed that the inner membrane of AC is covered by hyperplastic arachnoid cells, as well as cells in the cyst membrane – resembling foetal human arachnoid cells.

Using chemical analyses on AC fluid, Sandberg *et al.*[7] observed a composition comparable with cerebrospinal fluid (CSF). In addition, they found that some cysts had elevated protein levels in their fluid, relative to reference values. In a previous study on AC fluid and CSF from the same patient population as the current study, Berle *et al.*[4] found AC fluid and CSF to be similar in electrolyte content, except for an increased phosphate content in cyst fluid. The following components were reduced in AC fluid compared with CSF: 1) the total protein amount 2) lactate dehydrogenase and ferritin. Based on the decrease in protein concentration, we would suspect a molecular weight gradient, although the number of measured proteins was low.

Helland *et al.*[5] and Aarhus *et al.*[6] found differentially expressed mRNA and DNA copy number in AC membrane relative to normal arachnoid membrane for the genes NKCC1 [5] and ASGR1, DPEP2, SOX9, SHROOM3, A2BP1, ATP10D, TRIML1, BEND5 and NMU [6]. The NKCC1 is an active salt pump, that conceptually could contribute to the filling of AC. Zeuthen [13] discussed water transport in tissues against osmotic barriers and suggested that this transport is energised by ion transport, thus opening for active or selective transport as a filling mechanism. For active pumps such as the sodium – potassium – chloride transporter NKCC1, the amount of water co-transported is interesting - a single load of 1 Na^+^, 1 K^+^, 2 Cl^-^ may co-transport as much as 590 H_2_0-molecules.

In the same patient population as the current study, Berle *et al.*[11] performed a qualitative proteomics study of AC fluid, where the 199 identified proteins in a pool from 11 individual patients did show a similar protein expression as in normal CSF (195/199 proteins), dissimilar from that of plasma. A qualitative protein comparison study between 14 patients did indicate AC fluid protein profiles to be relatively homogenous between patients. Label-free quantitative proteomics by measured precursor intensity is a well-established method for obtaining relative quantitative measurements of a large number of proteins between samples [14]. Quantitative proteomics by Isobaric tag for relative and absolute quantification (iTRAQ) [15] allows for extensive fractionation at the peptide level of samples pooled after labelling without loosing analytical reproducibility. The possibility for extensive fractionation makes it possible to identify and quantify a larger portion of the proteome. This method requires a significantly larger amount of sample material. The quantitative proteomics methods applied here have been described thoroughly elsewhere [16].

In this study we used proteomics to quantitatively compare the protein content of AC fluid and CSF from the same patients, in order to identify possible differences in the proteomes between these two fluids. Two different approaches were undertaken; one where samples from individual patients were analysed using a label-free approach, and one where individual patient samples were pooled, iTRAQ-labelled and extensively fractionated to allow for a more in-depth quantitative analysis of the proteomes. The two complementary proteomics approaches were expected to give us a better insight in the content of AC fluid and potentially the mechanisms of fluid filling. Furthermore, we wanted to use the quantitative data to evaluate previously published results on AC, both to test the hypothesis of a molecular weight gradient over the AC membrane, and the comparison to previous published DNA and mRNA-results.

## Materials and methods

### Participants

Patients were recruited by the responsible surgeon and had signed a written informed consent. This project was approved by the Regional Committee for Medical and Health Research Ethics (REK) of Western Norway (approvals REK 70.03, NSD 9634 and REK 2009/1885).

Five participants, two males, three females, age 22–60 years, with unilateral temporal ACs were included in this study. AC fluid was collected during decompressive cyst surgery at Haukeland University Hospital (Bergen, Norway). The samples were selected from our biobank that contains samples of AC fluid and CSF from the same patients. All the patients fulfilled the following criteria: no previous intracranial surgery, no intracranial bleeding or trauma, and relatively low intraoperative blood contamination in both AC fluid and CSF as estimated from visual inspection and measured by mass spectrometry. An overview of the patients included is presented in Table 1.

**Table 1:** Age, gender and protein concentration in AC fluid and CSF for the included patients

**Patient number**	**Age**	**Gender**	**Protein concentration AC fluid μg/μl**	**Protein concentration CSF μg/μl**
1	26	f	0.44	0.57
2	43	m	0.38	0.62
3	22	f	0.31	0.59
4	41	f	0.29	0.59
5	60	m	0.50	0.59
**Average**			0.38	0.59
**protein content ratio AC fluid/CSF**				0.65

### Sample collection

The methods for sample collection and handling protocol as well as the laboratory work-up used in this study have previously been described in detail [4,17]. Briefly, AC fluid was collected during elective surgery for AC (craniotomy with fenestration and extirpation of the cyst) by puncturing the dura with a 23G, 25 mm long syringe needle using an Optidynamic® spinal fluid manometer (Mediplast AB, Malmo, Sweden) as siphon, through a burr hole before the craniotomy and opening of the dura. The fluid was centrifuged at 450*× g* for 5 min to remove cells and cell debris, and the supernatant was aliquoted and frozen at −80°C. Deviations and observations on individual sample material were noted on sampling.

After opening the medial cyst membrane that covered the basal structures (tentorial slit, oculomotor nerve, carotid artery, and the optic nerve), thus creating communication to the basal cisterns and the posterior fossa, a CSF-sample was collected with a pre- cut baby-feeding catheter #6, connected to a 10 ml syringe. The catheter was placed below the tentorium via the tentorial slit and fluid was aspirated gently from the posterior fossa. The collected CSF was processed in the same manner as the cyst fluid.

### Chemicals

Trypsin was purchased from Promega (Fitchburg WI, USA). N-octyl-β-D-glycopyranoside (NOG) was purchased from Anatrace (Maumee, OH, USA). Urea, acetonitrile (ACN), formic acid (FA), calcium chloride (CaCl2), iodoacetamide (IAA) and dithiothreitol (DTT), potassium phosphate monobasic (KH2PO4), potassium chloride (KCl), water and trifluoroacetic acid (TFA) were purchased from Sigma-Aldrich (St. Louis MO, USA). Water and ACN were of HPLC quality.

### Label free sample preparation

**Figure 1:**
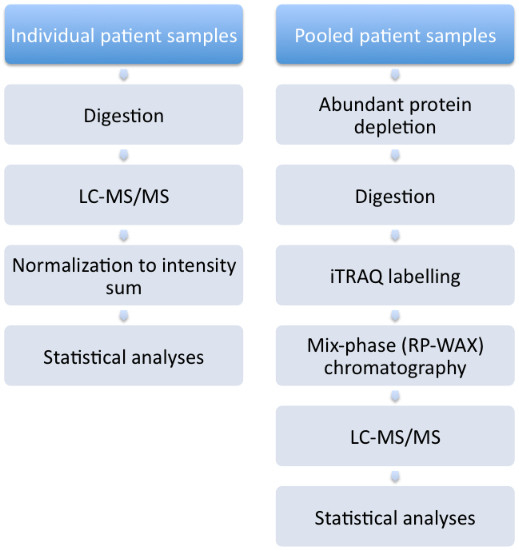
Overview of procedures undertaken.

A flow chart of the label-free proteomics experiment comparing the protein content between AC fluid and CSF for five individual patients is given in Figure 1. The protein concentration in AC fluid and CSF was measured using a QubitTM fluorometer (Life Technologies, Carlsbad CA, USA). The individual AC fluid and CSF samples were concentrated and desalted using Amicon 3 kDa molecular weight cut-off filters (Millipore, Billerica, MA, USA) and dried in a vacuum concentrator (Eppendorf, Hamburg, Germany). The proteins were digested into peptides by trypsin as described [18], a brief summary follows: the dried protein pellet was dissolved in 6 M Urea and 100 mM DTT and incubated for 1h at room temperature (RT). Cysteins were alkylated using 200 mM iodoacetamide during one hour incubation at RT. Chymotrypsin activity was inhibited by adding 2 mM CaCl2 and the proteins were digested to peptides over night at 37°C using trypsin (Sequencing Grade Modified Trypsin, Promega) at a protein:trypsin ratio of 1:50. Each sample was acidified using 10% FA to quench the digestion activity, desalted and concentrated on an Oasis HLB μElution Plate (Waters) as previously described [14] followed by drying the sample completely in a vacuum concentrator.

### Sample preparation prior to iTRAQ-labelling

A flow chart of the iTRAQ proteomics experiment comparing the protein content between AC fluid and CSF pooled from five patients is given in Figure 1. AC fluid (150 μl) from each of the five patients was used to generate a pool of 750 μl, and a pool of CSF was made in the same way for the same patients. 250 μl of each of these two pools were combined into a mix-pool. 500 μl from each of the AC fluid pool, CSF pool and mix-pool was concentrated using 3 kDa ultracentrifugation filters (Amicon Ultra-4, Millipore, Bedford, MA), which were pre-rinsed with 0.1% NOG. The samples were then depleted of high abundant proteins using a human Multiple Affinity Removal System (MARS Hu-14) 4.6 mm × 50 mm LC column (Agilent Technologies) according to the protocol provided by the supplier, using a Dionex 3000-series LC system. This column depletes albumin, IgG, antitrypsin, IgA, transferrin, haptoglobin, fibrinogen, alpha-2-macroglobulin, alpha-1-acid glycoprotein, IgM, apolipoprotein AI, apolipoprotein AII, complement C3, and transthyretin. The high-abundant protein-depleted samples were concentrated using 3 kDa ultracentrifugation filters which were pre-rinsed with 0.1% NOG. Each sample was freeze-dried prior to protein digestion and iTRAQ labelling.

### Protein digestion and iTRAQ-labelling

For the pooled patient samples, the total protein in each depleted sample was reduced, alkylated, digested with trypsin, and iTRAQ-labelled according to the manufacturer‘s protocol using the reagents provided (Applied Biosystems, Foster City, CA). The reduced and S-methylmethanethiosulfonate (MMTS)-treated proteins were digested to peptides over night at 37°C using 2.5 μg trypsin as protease. The peptides were iTRAQ-labelled (4plex) where the mix-pool 50:50 AC fluid and CSF with the 114 label, the AC fluid with the 115 label, and CSF with the 116 label. All samples were combined after the labelling was conducted.

### Mix-phase chromatograpy

iTRAQ labelled peptides were fractionated in 28 fractions using mix-phase chromatography utilizing a Sielc Promix column (MP-10.250.0530, 1.0 × 250 mm, 5 μm, 300Å, Sielc Technologies, Prospect Heights, Illinois), using an Agilent 1260 series LC system (Agilent Technologies, Palo Alto, CA). The peptides were reconstituted in buffer A (20 mM ammonium formate, 3% ACN) and loaded on the Mix phase column using 85% A for 10 min at a flowrate of 50 μl/min. The peptides were eluted using a gradient of 15% - 60% buffer B (2 mM ammonium formate, 80% ACN, pH 3.0) over 35 min, 60%-100% B over 10 min and held constant for 5 min. The fractions were collected every 2 min until 60 min, the last 10 min of the LC run was collected in 2 fractions of 5 min. The fractions from the 8 first min of the gradient were discarded.

### Orbitrap mass spectrometry

#### Injection and LC

For the label-free experiment, 0.5μg (as determined by the protein concentration measurement) of each peptide sample dissolved in 1% aqueous formic acid, was injected into an Ultimate 3000 RSLC system (Thermo Scientific, Sunnyvale, California, USA) connected online to a linear quadrupole ion trap-Orbitrap (LTQ-Orbitrap Velos Pro) mass spectrometer (Thermo Scientific, Bremen, Germany) equipped with a nanospray Flex ion source (Thermo Scientific). The sample was loaded and desalted on a pre-column (Acclaim PepMap 100, 2 cm × 75 μm i.d. nanoViper column, packed with 3μm C18 beads) at a flow rate of 5μl/min for 6 min using an isocratic flow of 0.1% FA (vol/vol) with 2% ACN (vol/vol).

Peptides were separated during a biphasic ACN gradient from two nanoflow UPLC pumps with flow rate of 280 nl/min on the analytical column (Acclaim PepMap 100, 15 cm x 75μm i.d. nanoViper column, packed with 2μm C18 beads). Solvent A was 0.1% FA (vol/vol) with 2% ACN (vol/vol). Solvent B was 0.1% FA (vol/vol) with 90% ACN (vol/vol). The gradient composition was 5–38% B from LC starts to 67 min, then 38–90% B from 67–70 min. 90% B was held constant for 5 min, followed by column conditioning for 12 min with 5% B.

### Individual patient samples

The eluting peptides were ionised in the electrospray and analyzed by the LTQ-Orbitrap Velos Pro. The mass spectrometer was operated in the DDA-mode (data-dependent-acquisition) to automatically switch between full scan MS and MS/MS acquisition.

Survey full scan MS spectra (from m/z 300 to 2000) were acquired in the Orbitrap with resolution R = 60000 at m/z 400 (after accumulation to a target value of 1e6 in the linear ion trap with maximum allowed ion accumulation time of 500ms). The seven most intense eluting peptides above an ion threshold value of 1000 counts, and charge states 2 or higher, were sequentially isolated to a target value of 1e4 and fragmented in the high-pressure linear ion trap by low-energy CID (collision-induced-dissociation) with normalised collision energy of 40% and wideband-activation enabled. The maximum allowed accumulation time for CID was 200ms, the isolation width maintained at 2Da, activation q = 0.25, and activation time of 10ms. The resulting fragment ions were scanned out in the low-pressure ion trap at normal scan rate, and recorded with the secondary electron multipliers. One MS/MS spectrum of a precursor mass was allowed before dynamic exclusion for 30s. Lock-mass internal calibration was not enabled.

### Pooled patient samples

The settings were identical to those mentioned above for CID fragmentation, with the exception that the seven most intense eluting peptides were sequentially isolated in the high-pressure linear ion trap by low-energy CID and in the octopole HCD collision cell by HCD (Higher Energy Collision Dissociation) fragmentation. Both fragmentation forms used normalised collision energy of 40%. For HCD fragmentation the isolation width was 3Da and the activation time 0.10ms. After fragmentation in the HCD cell the fragments were transferred via the C-trap to the Orbitrap and scanned out with resolution R = 7500. One MS/MS spectrum of a precursor mass was allowed before dynamic exclusion for 20s.

### Data analysis

The handling of multiple testing and validation of peptide and protein hits were assessed by false discovery rate (FDR) [19]. The data from the individual patients’ label-free experiment were compared by paired 2-sided *t*-test with p < 0.05.

The individual patient data obtained from the label-free quantitative analysis was compared using Progenesis LC-MS (Nonlinear Dynamics Ltd. Newcastle upon Tyne, UK) (v4.0.4573.30654). The data was searched against the SwissProtKB database (release 2011_10) using SearchGUI (1.8.3) with OMSSA and XTandem as search engines and PeptideShaker (0.16.2) (http://code.google.com/p/peptide-shaker/) for combining the results [20]. The following search criteria were used: fixed modifications carbamidomethylation, oxidated methionine as variable modification, a maximum of two missed cleavages, precursor mass tolerance 15 ppm, and product mass tolerance 0.7 Da. The peptides were auto-validated with maximum FDR of 1.0%. The quantitative data obtained from the individual patients in the label-free experiment was compared by a paired 2-sided *t*-test. Proteins with a p-value of less than 0.05 combined with an average fold change of more than +/− log2 (0.58) were considered as differentially abundant between CSF and AC fluid.

The pooled patient iTRAQ-data was searched using the Spectrum Mill software package v4.0 (Agilent Technologies, Santa Clara, CA) using the same settings as previously described [14]. MS/MS data was searched against the SwissProtKB database (release 2011_10) with a precursor mass tolerance of 15 ppm, and a product mass tolerance 0.7 Da. The peptides were validated by auto-determined score by delta R1-R2 threshold with max FDR of <1.2%. Proteins were validated by minimum protein score of 20. To normalize the data, we re-centred the protein ratios of each sample versus the reference sample by subtracting the median in each iTRAQ channel from each ratio so that the distribution was symmetrical around a log2 ratio of 0, as described elsewhere [17]. The reference/CSF value was divided by the reference/AC fluid value for every protein to obtain a CSF-to-AC fluid ratio. All proteins with a fold change more than +/− log2 (0.58) were considered as differentially abundant between CSF and AC fluid.

Using the bioinformatics software J-Express Pro 2.7 (MolMine AS, Bergen, Norway) proteins with increased or reduced abundance between CSF and AC fluid in both label-free and iTRAQ labelled experiments were compared with the corresponding gene expression profiles of a previously published study [6]. The objective of this examination was to evaluate whether AC fluid protein expression could be linked to membrane mRNA expression. The proteins were searched in PubMed for official gene symbol, and then searched against the microarray data to obtain the corresponding mRNA expression profile.

Gene ontology data for the identified proteins in the iTRAQ experiment were obtained using ProteinCenter version 3.9.10025 (Thermo-Fischer scientific, Odense, Denmark).

## Results

**Figure 2:**
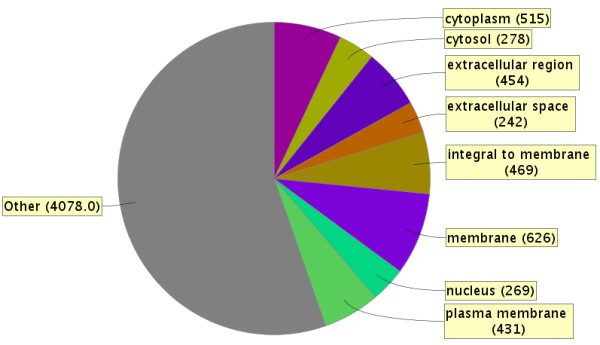
**Genetic Ontology of cellular localization of the 1425 quantified protein groups in the iTRAQ-labeled pooled patient samples.** Figure made utilizing ProteinCenter version 3.9.10025.

In the quantitative individual label-free comparison, we quantified 348 proteins, of which 150 were differentially expressed between AC and CSF (p < 0.05 in a paired two-sided *t*-test, minimum 2 peptides, FDR < 1.0%). In the iTRAQ-labelled quantitative experiment, 1425 proteins were identified (minimum 2 peptides, FDR <1.2%). 296 protein groups were identified both in the label-free and the iTRAQ-labelled experiment. The list of proteins identified from the quantitative iTRAQ-labelled experiment, ranked by fold change AC/CSF, is presented in Additional file 1: Table of Proteins identified in the iTRAQ-labelled experiment. Proteins identified in the label-free experiment with individual and average fold change, p-value, and corresponding iTRAQ fold change value (when applicable) are shown in Additional file 2: Table of proteins identified in the label-free experiment. The iTRAQ quantification experiment is also the single largest qualitative characterization of AC fluid proteins. The 1425 proteins identified and quantified in the iTRAQ-experiment were annotated to a multitude of cellular localizations, of which the principal classes are presented in Figure 2. A high proportion of the proteins were annotated to extracellular or membrane space. Note that one protein may be allocated to several localizations and may therefore be counted more than once.

We found 22 proteins with significantly higher abundance in the AC fluid relative to CSF and 24 proteins with significantly lower abundance (Table 2). These proteins were selected on significant differential abundance by *t*-test between individual samples, as well as fold change +/− log2 (0.58) in both individual label-free samples and iTRAQ-labelled samples. Concerning the proteins with changed abundance in AC fluid relative to CSF, we observed no specific pattern in the type of proteins. Some, but not all, of the proteins with reduced abundance in AC relative to CSF are typical blood proteins. Examples of such proteins were carbonic anhydrase 1, fibrinogen, alpha-1-antitrypsin and haemoglobin. Carbonic anhydrase 1 has previously been reported to be present in CSF due to contamination of blood introduced during sample collection [21].

**Table 2:** Overview of the quantitative protein results from the comparison of the two experiments

**Accession number**	**Protein name**	**iTRAQ unique peptides**	**Label-free unique peptides**	**p**	**iTRAQ ratio**	**Individual average fold change**
O94772	Lymphocyte antigen 6H	4	3	0,023	9,7	1,5
P13987	CD59 glycoprotein	4	3	0,033	5,6	1,6
P16070	CD44 antigen	4	3	0,026	2,3	2,0
P55058	Phospholipid transfer protein	17	7	0,026	2,2	2,2
P01033	Metalloproteinase inhibitor 1	7	4	0,019	2,0	2,0
O00584	Ribonuclease T2	14	3	0,010	2,0	2,3
P02747	Complement C1q subcomponent subunit C	2	4	0,013	1,9	1,4
P48745	Protein NOV homolog	9	3	0,011	1,9	1,7
P07602	Proactivator polypeptide	15	9	0,042	1,9	1,7
P10909	Clusterin	25	26	0,001	1,8	1,9
Q969P0	Immunoglobulin superfamily member 8	8	4	0,008	1,7	1,7
Q9Y6R7	IgGFc-binding protein	27	9	0,002	1,7	2,2
Q6UX71	Plexin domain-containing protein 2	14	3	0,008	1,6	1,8
Q02246	Contactin-2	35	19	0,011	1,6	2,2
P04066	Tissue alpha-L-fucosidase	11	2	0,009	1,6	2,7
Q96GW7	Brevican core protein	27	11	0,016	1,6	1,6
P08253	72 kDa type IV collagenase	31	5	0,005	1,6	1,9
Q15113	Procollagen C-endopeptidase enhancer 1	16	10	0,010	1,5	1,8
P08571	Monocyte differentiation antigen CD14	14	9	0,000	1,5	1,6
O75509	Tumor necrosis factor receptor superfamily member 21	7	3	0,018	1,5	1,6
P20062	Transcobalamin-2	11	2	0,036	1,5	2,9
Q12860	Contactin-1	44	18	0,003	1,5	1,6
P60174	Triosephosphate isomerase	15	8	0,020	0,6	0,4
Q99497	Protein DJ-1	15	4	0,036	0,5	0,4
P19827	Inter-alpha-trypsin inhibitor heavy chain H1	19	7	0,032	0,5	0,3
P19823	Inter-alpha-trypsin inhibitor heavy chain H2	24	7	0,032	0,5	0,4
P06744	Glucose-6-phosphate isomerase	16	3	0,034	0,4	0,3
P09382	Galectin-1	5	4	0,041	0,4	0,4
P62258	14-3-3 protein epsilon	16	4	0,032	0,3	0,2
P63104	14-3-3 protein zeta/delta	14	3	0,027	0,3	0,3
P04040	Catalase	18	2	0,001	0,3	0,2
P62937	Peptidyl-prolyl cis-trans isomerase A	11	4	0,022	0,3	0,2
P09211	Glutathione S-transferase P	9	6	0,029	0,3	0,3
P32119	Peroxiredoxin-2	12	4	0,001	0,3	0,1
P00558	Phosphoglycerate kinase 1	25	4	0,036	0,3	0,3
P02675	Fibrinogen beta chain	16	12	0,003	0,3	0,5
P08107	Heat shock 70 kDa protein 1A/1B	27	5	0,034	0,3	0,2
P02671	Fibrinogen alpha chain	14	8	0,001	0,3	0,5
P63261	Actin, cytoplasmic 2	21	5	0,020	0,2	0,1
P07437	Tubulin beta chain	8	2	0,033	0,2	0,2
P02679	Fibrinogen gamma chain	5	10	0,001	0,2	0,5
P02042	Hemoglobin subunit delta	14	7	0,008	0,2	0,1
P00915	Carbonic anhydrase 1	9	5	0,004	0,2	0,0
P68871	Hemoglobin subunit beta	14	12	0,006	0,2	0,0
P08670	Vimentin	33	27	0,027	0,2	0,1
P69905	Hemoglobin subunit alpha	10	13	0,003	0,1	0,0

Proteins with different abundance between AC fluid and CSF, determined by significant (p < 0.05) differential abundance in individual label-free samples, as well as fold change +/− log2 (0.58) in both label-free and iTRAQ experiment.

Among the proteins being more abundant in AC fluid was ribonuclease T2 (Gene name: RNASET2). Defects in RNASET2 are proposed to be the cause of cystic leukoencephalopathy without megalencephaly (LCWM), and the brain of such affected individuals shows anterior temporal lobe subcortical cysts.

We were not able to link the molecular functions of the 46 differentially abundant proteins to the cyst fluid biology based on the examined GO functional terms and Uniprot functional annotation (data not shown). The cellular location of the differentially abundant proteins between AC fluid and CSF were mainly membrane and secreted proteins as seen from the GO analysis (data not shown).

In the iTRAQ quantification, we identified 1129 proteins that were not present in the list from the label-free experiment. The use of depletion for the most abundant proteins as well as more extensive fractionation caused this increase in the number of proteins quantified. We observed that 480 of the proteins quantified in the iTRAQ experiment were outside the selected boundaries of significant fold change (+/− log2 (0.58)), but we cannot from this experiment conclude if any of these proteins represent a true biological change in abundance without additional verification. We observed that these 480 proteins to a lesser degree seem to represent membrane or extracellular proteins, relative to the proteins reported as differential abundant in both experiments (data not shown).

From the 46 proteins we identified as differentially abundant in our study, 45 were also detected from the mRNA expression level study. None of them were reported as differentially expressed [6]. We identified no specific patterns of altered abundance between the membrane mRNA and the 46 differentially abundant cyst fluid proteins (Additional file 3: Comparison of protein results versus mRNA data.). We did not quantify any of the proteins associated with the ten genes previously reported as differentially abundant between AC membrane and AM [5,6] (data not shown).

To test the hypothesis of molecular weight gradients over the AC membrane, we created a scatter plot for all the iTRAQ protein ratios sorted based on protein molecular weight. We could not see any correlation between increased molecular weight and decreased abundance in AC compared to CSF from this plot (results not shown).

## Discussion

In this study, we identified by far the largest number of AC fluid proteins ever reported, and this is also the first large-scale quantitative comparison between the protein content of AC fluid and CSF collected from the same patients. At present, there are three dominating hypotheses on the mechanisms of filling and sustaining of AC: secretion or selective transport, oncotic filling, and a slit-valve mechanism. Previous reports of reduced protein content in AC fluid relative to CSF [4,7] in the same sample population as the current study do weaken the hypothesis of filling by oncotic pressure.

From our two quantitative proteomics experiments, we identified 46 proteins with altered abundance between AC fluid and CSF. Some of the protein groups with lower abundance in AC fluid seem to have a high representation of blood proteins. Blood contamination is an obvious problem when sampling is not identical between the sample types to be compared, in this case AC fluid and CSF. Because we sample AC fluid through a syringe needle after a direct puncture of the cyst, the risk for blood contamination of the cyst fluid is low. CSF is collected in the basal cistern after the operation is finished and haemostasis is ensured. This might lead to a small and variable contamination of blood in the CSF samples. Hence, abundance changes in these typical blood proteins are probably not representing AC biology, but are rather introduced to CSF during sample collection. Currently, there is no consensus on how to handle skewed blood contamination. Most of the proteins with reduced abundance in AC relative to CSF are however not termed “blood specific” proteins.

A challenge in the evaluation of quantitative difference is the defining criteria on what is differentially abundant protein. In our case, we choose to include proteins that were observed with fold change above +/− log2 (0.58) in both experiments, as well as a p-value below 0.05 for the label-free experiment. This result might be somewhat conservative for exploratory analyses; hence we also evaluated the abundance changes for the 1129 proteins only quantified in the iTRAQ study.

The 480 proteins from the iTRAQ study with fold change above +/− log2 (0.58) in this analysis does however need further verification in different sample sets, by different methods or specific validation by for example selective reaction monitoring to increase the certainty of these findings. Further verification is also necessary to confirm the differential abundance of the 46 proteins found as differentially abundant in both experiments in a larger number of patients. Our current results give however support to the previous claim [4] that AC fluid is different from CSF. As observed from our Qubit protein measurements, the general trend of reduced total protein concentration in AC fluid relative to CSF does not support oncotic pressure gradients. Observing differential abundance of proteins between AC fluid and CSF, as well as the lack of observed slit valves in general in the literature, do in principle not support the theory of valve mechanism. In case of a valve mechanism, we would expect a higher similarity in the quantitative proteomes between AC fluid and CSF than what we observe to support this theory. In addition, to the authors’ knowledge, slit valves have only been observed in suprasellar AC and not in temporal AC. We therefore believe that such valve mechanisms are less likely to be responsible for AC filling, but we do not have definitive evidence against it. Differential abundance of proteins between AC fluid and CSF is supportive of some kind of secretion or selective transport, but we are not able to elute which on basis of our results.

Previous reports have identified an up-regulation of mRNA for several ion transporters [5] and other genes [6] in AC membrane when compared with normal arachnoid membrane. In our proteomics study, we hypothesised that some of the proteins in the AC membrane corresponding to these mRNA transcripts also could be found in the AC fluid, but not to the same extent in CSF, and that such proteins possibly could give an indication of the mechanism of transport over the membrane. However, we were not able to draw such lines based on our obtained data. Concerning the proteins that make up active pumps, such as NKCC1, they would be a part of the AC wall and thus not necessarily detectable in AC. A lack of confirmation in our data does therefore not exclude that such pumps can be found in the AC membrane, in particular since hydrophobic membrane proteins might not at all be detectable in the AC fluid due to solubility issues.

In a previous study, Berle *et al.*[4] suggested a MW gradient from the reported reduced concentration of macromolecules ferritin and lactate dehydrogenase. The extended examinations of the data presented in the current work, with the lack of correlation between quantitative ratios and molecular weight, contradict such a hypothesis of MW gradients over the AC membrane.

## Conclusions

From our experiments, the protein content of AC fluid and CSF appears to be very similar. Some proteins were, however, significantly differentially abundant between AC fluid and CSF. This could reflect that these proteins are affected by the filling mechanism of arachnoid cysts or are shed from the membranes into arachnoid cyst fluid, but we are not able to identify the mechanism. We did not identify protein products in AC fluid from the previously suggested pumps found in the AC membrane or specific MW gradients in distribution of proteins between AC fluid and CSF. Our results do not support the mechanisms of oncotic pressure or valves. Based on these results we suggest that some sort of secretion or selective transport causes AC filling across the AC membrane.

## Abbreviations

AC: Arachnoid cyst; ACN: Acetonitrile; CID: Collision-induced dissosiation; CSF: Cerebrospinal fluid; DTT: dithiothreitol; FA: Formic acid; FDR: False discovery rate; HCD: Higher Energy Collision Dissociation; HPLC: High pressure liquid chromatography; IAA: Iodoacetamide; iTRAQ: Isobaric tag for relative and absolute quantification; LTQ: Linear trap quadropole; MW: Molecular weight; NOG: N-octyl-β-D-glycopyranoside; NSE: Gamma-enolase; PPM: Parts per million; REK: Regional Committee for Medical and Health Research Ethics; TFA: Trifluoroacetic acid.

## Competing interests

The authors declare that they have no competing interests.

## Authors’ contributions

MB, ACK, HG, MA, OAH, KW, RJU, CAH, FSB conceived and designed the experiments. ACK, HG, performed the experiments. MB, ACK, HG, MA, OAH, FSB analyzed the data. KW, CAH operated on the patients. MB performed sample collection and handling. MB, ACK, HG, MA, KW, CAH, FSB wrote the paper. All authors read and approved the final manuscript.

## Supplementary Material

Additional file 1 **Table of proteins identified in the iTRAQ-labelled experiment.** The proteins are ranked by fold change between AC fluid and CSF. Click here for file

Additional file 2 **Table of proteins identified in the label-free experiment.** The proteins are reported with individual and average fold change, p-value and corresponding iTRAQ fold change value (when applicable). Click here for file

Additional file 3 **Comparison of protein results versus mRNA data.** The reported proteins with increased or decreased abundance in AC fluid relative to CSF are plotted against corresponding membrane mRNA microarray data in order of significance in membrane data. Click here for file
